# A longitudinal psychological model of writing development: interactions among self-efficacy, regulation, and anxiety

**DOI:** 10.3389/fpsyg.2026.1757045

**Published:** 2026-02-19

**Authors:** Sijia Cheng, Chenming Lin, Dan Wei, Yu Chen

**Affiliations:** 1 School of International Education, Anhui University of Technology, Anhui, China; 2 Department of Foreign Languages, Tongji Zhejiang College, Zhejiang, China; 3 Faculty of Social Sciences and Liberal Arts, UCSI University, Kuala Lumpur, Malaysia; 4 School of Foreign Languages, Southeast University, Nanjing, China

**Keywords:** EFL learners, self-regulated writing strategy, writing anxiety, writing performance, writing self-efficacy

## Abstract

This longitudinal study investigates the temporal relationships among writing self-efficacy, self-regulated writing strategies, writing anxiety, and writing performance in English-as-a-Foreign-Language (EFL) contexts. Guided by Social Cognitive Theory (SCT), Self-Regulated Learning (SRL) Theory, and Cognitive Load Theory (CLT), this study examines how motivational beliefs and regulatory behaviors interact over time to shape writing outcomes. Data were collected from 468 Mandarin-speaking EFL learners across three waves (T1–T3) over a 12-week semester, using validated instruments for self-efficacy, self-regulated strategies (cognitive, metacognitive, motivational regulation, and social behavior), and multidimensional writing anxiety, alongside a standardized performance assessment. Longitudinal structural equation modeling revealed that earlier writing self-efficacy was positively associated with subsequent use of self-regulated writing strategies, which in turn predicted lower levels of later writing anxiety and higher writing performance. Among strategy dimensions, metacognitive strategies exhibited the strongest mediating role, while somatic anxiety emerged as the most performance-impairing factor. Gender-based analyses indicated that male learners relied more on social-behavioral strategies, whereas female learners benefited more from metacognitive regulation. These findings advance theoretical understanding by demonstrating how belief–strategy–affect linkages unfold over time, providing empirical support for an integrative SCT–SRL–CLT framework. Pedagogically, the study underscores the importance of sustained instruction that strengthens self-efficacy, fosters metacognitive strategy use, and systematically manages writing-related anxiety to enhance long-term writing development in EFL learners.

## 1 Introduction

There is an increasing agreement among scholars that writing skills are for adolescent learners a prerequisite for success in the 21st century (Mirra and Garcia, 2021). Writing is perceived as a fundamental ability that transcends the traditional boundaries of academia, shaping our ability to communicate effectively, think critically, and solve problems creatively throughout various aspects of life (Liu et al., 2024). As a result, many employers and higher education institutions expect students to have advanced first language (L1) literacy skills that prepare them to write academic essays, technical reports, and professional papers (Lindström and Eklund Heinonen, 2024). As we move forward into a knowledge economy, composing written communication is now one of the most basic elements of professional success, and consequently, writing skills have become one of the critical factors in hiring and career advancement. Moreover, the importance of written communication extends beyond the language of communication itself. In some education systems, national language education policies require students to study and develop literacy skills in more than one language during primary education (Lee and Phua, 2020; Sim, 2016). Second language (L2) and foreign language (FL) proficiency have a substantial impact on students’ academic and career trajectories. Tai and Zhao (2024) found that L2 writing proficiency plays an important role when it comes to students’ access to higher education institutions and for opportunities to work and develop in their careers. Many universities and employers assess applicants’ writing skills as part of their selection process, which emphasizes the importance of academic and professional writing skills in multiple languages.

EFL learners have several difficulties related to their linguistic knowledge when engaged in L2 writing including insufficient vocabulary, grammatical errors and issues with syntactic complexity (Li and Akram, 2024). They might also have difficulty with writing skills in a more global sense, such as organization, idea development, and cohesion (Aydın and Yüce, 2024). These challenges are compounded by affective burdens, like writing anxiety, which surfaces as apprehension, stress, or fear in relation to writing tasks. Heidarzadi et al. (2022) conceptualized L2 writing anxiety as a multidimensional construct including cognitive anxiety, somatic anxiety, and avoidance behavior, which is often associated with learners’ concerns about linguistic performance and fear of negative evaluation in writing contexts. Such feelings of inadequacy can negatively influence learners’ motivation, interest, and willingness to write and affect their performance.

Mostly, L2 writing success depends on individual learner factors like anxiety, self-efficacy, etc. (Yeung et al., 2024). Anxiety, if not addressed appropriately, can serve as an obstacle to the thought process of combining all skills and knowledge of the student during writing. On the other hand, self-efficacy (the belief in one’s ability to achieve specific goals) has been documented to have a positive effect on students’ persistence, effort, and ability to leverage writing challenge (Shen et al., 2024). Learners who have high levels of self-efficacy can help themselves feel more confident and feel more in control when they are writing and are able to think of ways to enhance their skills even when they are having difficulties.

Building on these individual learner factors, self-regulated writing strategies have recently emerged as a focal point of research in EFL contexts, primarily because of their potential to significantly enhance both writing performance and learner autonomy (Fan and Wang, 2024). These strategies empower learners to take control of their writing processes by planning, monitoring, and reflecting on their tasks, thereby fostering a more proactive and strategic approach to learning. Numerous studies have demonstrated that learners who employ effective self-regulated strategies tend to perform better in writing tasks, as these strategies help them manage cognitive and emotional challenges, organize their ideas, and produce coherent and well-structured texts (Teng and Huang, 2019). Moreover, self-regulated writing strategies have been found to interact closely with psychological constructs such as self-efficacy (Shen and Bai, 2024).

Taken together, recent empirical research has increasingly converged on the view that L2 writing performance is shaped by the dynamic interaction of individual learner factors and self-regulatory processes. In particular, studies have consistently highlighted the central roles of writing self-efficacy, writing anxiety, and self-regulated writing strategies in explaining individual differences in writing engagement and performance in EFL contexts (e.g., Fan and Wang, 2024; Shen et al., 2024; Teng and Huang, 2019; Yeung et al., 2024). These findings suggest that successful L2 writing development depends not only on linguistic competence but also on learners’ ability to regulate cognitive and emotional demands throughout the writing process.

Accordingly, the present study is theoretically grounded in SCT, SRL Theory, and CLT, which together provide a framework for understanding how motivational beliefs, regulatory strategies, and affective–cognitive constraints jointly shape L2 writing performance. A detailed theoretical elaboration of these frameworks is presented in the following Literature Review section.

## 2 Theories and literature review

### 2.1 Writing self-efficacy

Self-efficacy, characterized as an individual’s conviction in their capacity to excel in particular tasks, is an essential psychological construct that profoundly impacts learning and performance in educational settings (Li et al., 2024). In writing, self-efficacy significantly influences learners’ attitudes, motivation, and engagement with the writing process (Wang et al., 2024b). Writing self-efficacy denotes a learner’s confidence in their capacity to effectively execute diverse writing activities, such as idea generation, content organization, and compliance with language rules. This conviction is crucial as writing is a cognitively intensive and complex activity that demands perseverance, concentration, and flexibility. A study by Kim et al. (2024) determined that students with elevated writing self-efficacy are more inclined to undertake demanding writing assignments, persist in the face of obstacles, and proactively explore methods to enhance their writing skills. In EFL environments, where learners frequently encounter supplementary language and cultural obstacles, writing self-efficacy is crucial since it alleviates the feelings of frustration and helplessness that may stem from these impediments (Chen and Hsu, 2022).

Empirical evidence consistently suggests that self-efficacy in writing tends to mitigate writing anxiety, a prevalent emotional obstacle encountered by learners, especially in EFL contexts. Writing anxiety denotes the unease and tension linked to writing assignments, frequently arising from the dread of adverse judgment, diminished self-assurance, or the view of writing as a high-pressure endeavor (Busse et al., 2023). Various studies have shown that individuals with elevated writing self-efficacy generally encounter reduced writing anxiety. For instance, Li (2022) discovered that self-efficacy mitigates writing-related stress by imparting a sense of control over the writing process to students. Similarly, Shen et al. (2024) highlighted that learners possessing strong writing self-efficacy are more adept at navigating the emotional hurdles associated with writing, as they tackle assignments with an optimistic outlook and prioritize problem-solving over the apprehension of failure. In EFL situations, where learners frequently contend with linguistic and cultural unfamiliarity, the correlation between self-efficacy and anxiety is notably significant (Sari and Han, 2024). Elevated self-efficacy assists learners in reinterpreting their views on writing obstacles and facilitates the adoption of appropriate coping methods, including the establishment of realistic objectives and the implementation of self-regulated techniques (Khosravi et al., 2023). Consequently, cultivating writing self-efficacy is a crucial element in alleviating writing anxiety and establishing a conducive atmosphere for language acquisition.

In addition to its impact on writing anxiety, writing self-efficacy is a significant predictor of writing performance. Students who possess confidence in their writing capabilities are more inclined to exert the necessary effort to attain superior outcomes. Shehzadi et al. (2021) and Abdi Tabari and Goetze (2024) indicated that self-efficacious learners engage in writing with enhanced motivation and perseverance, resulting in superior performance in both content and structure. Moreover, Sari and Han (2024) underscored that writing self-efficacy is positively associated with writing competency, as learners with elevated confidence are more inclined to effectively engage in planning, composing, and rewriting their work. In EFL situations, Abolhasani et al. (2022) and Göncü and Mede (2022) emphasized that writing self-efficacy is crucial as learners face hurdles including restricted vocabulary, grammatical inaccuracies, and unfamiliar discourse rules. Research, including that of Fathi et al. (2021), Yeung et al. (2024), and Aydın and Yüce (2024) indicates that self-efficacious learners in EFL environments utilize more self-regulated methods, allowing them to surmount linguistic challenges and generate coherent, well-structured texts. These findings highlight the significant impact of writing self-efficacy on learners’ writing success. By cultivating a robust sense of self-efficacy, educators may assist learners in acquiring the confidence and abilities essential for excelling in writing, so improving their academic and career opportunities. Accordingly, the present study examines writing self-efficacy as a key motivational construct within a broader model of self-regulated writing strategies and writing anxiety.

### 2.2 Self-regulated writing strategy

Self-regulated writing methods are crucial instruments that enable learners to manage their writing processes, improving their capacity to address the complex requirements of writing assignments. These tactics empower learners to systematically plan, monitor, and reflect on their writing, enhancing their autonomy and efficiency in the learning process (Teng and Zhang, 2024). Self-regulated writing strategies are typically classified into three dimensions: cognitive, metacognitive, and social behavior strategies (Teng and Zhang, 2016). Cognitive techniques emphasize the pragmatic elements of writing, including concept organization, paragraph construction, and the application of suitable terminology and syntax (Teng and Zhang, 2018). Metacognitive methods encompass advanced cognitive skills that assist learners in organizing their writing activities, tracking their advancement, and assessing the quality of their results (Teng, 2024). Conversely, social techniques underscore the importance of connection and collaboration, including soliciting input from peers or educators, participating in conversations, and acquiring knowledge from social environments (Teng et al., 2022). Collectively, these aspects establish an all-encompassing framework for navigating the intricacies of writing. In EFL situations, when learners encounter supplementary language and cultural obstacles, self-regulated writing skills are essential as they empower students to tackle writing tasks with confidence, resilience, and adaptation (Fan and Wang, 2024).

A growing body of empirical research has demonstrated that the use of self-regulated writing strategies is associated with lower levels of writing anxiety. Writing anxiety frequently arises from a deficiency of control over the writing process, apprehension regarding negative assessment, and the pressure to achieve elevated standards (Arnawa and Arafah, 2023). Utilizing self-regulated writing strategies, learners can alleviate these pressures by deconstructing the writing process into manageable components and proactively tackling challenging aspects (Zhou and Hiver, 2022). Studies by Guo et al. (2021), Bai et al. (2024), and Vattøy and Gamlem (2024) imply that metacognitive methods, including goal-setting and self-monitoring, are notably beneficial in alleviating anxiety. Among them, Bai et al. (2024) discovered that EFL learners who regularly employed metacognitive strategies experienced reduced writing anxiety, as these tactics provided them with enhanced clarity and control over their writing assignments. Likewise, Vattøy and Gamlem (2024) believed that the implementation of social methods, like soliciting feedback and participating in collaborative writing, has demonstrated a reduction in the fear of negative evaluation by fostering a helpful and constructive learning atmosphere. Cognitive methods, including drafting and editing, assist learners in managing the cognitive load of writing, so alleviating the feelings of overload frequently associated with writing activities (Guo and Bai, 2022). Equipping learners with these skills, self-regulated writing strategies mitigate anxiety, allowing pupils to engage in writing with a more positive and proactive attitude.

Besides alleviating anxiety, self-regulated writing procedures are significant indicators of writing performance. These tactics equip learners with the means to enhance the quality, coherence, and efficacy of their writing. Cognitive methods, including concept organization and language refinement, directly enhance the technical elements of writing (Teng et al., 2022). Yousef jarrah et al. (2023) emphasized that learners who consistently employ cognitive methods are more inclined to generate well-organized and coherent texts. Metacognitive tools, including planning and self-assessment, empower learners to tackle writing projects with a defined framework and to enhance their work progressively (Teng and Qin, 2024). Rahimi and Fathi (2022) discovered a favorable correlation between metacognitive self-regulation and elevated writing performance, indicating that learners who plan and monitor their tasks are more adept at identifying deficiencies and improving their final results. Social methods are essential for enhancing performance, especially in EFL situations, where learners gain from feedback and collaborative learning (Sabti et al., 2019). Research, like that of Zhan and Xu (2025), indicates that learners who proactively solicit and integrate feedback from peers and instructors exhibit considerable enhancements in their writing quality. These findings underscore that self-regulated writing strategies are not only auxiliary tools but essential elements of writing success (Sabti et al., 2019). By implementing these tactics, instructors can assist learners in becoming more proficient, autonomous, and self-assured writers, so improving their academic and professional results. In this study, self-regulated writing strategy is therefore conceptualized as a central explanatory mechanism linking learners’ motivational beliefs, writing anxiety, and writing performance.

### 2.3 Theoretical framework

#### 2.3.1 Social cognitive theory (SCT) and writing self-efficacy

SCT (Bandura, 1986) frames self-efficacy as a central driver of human agency. Individuals’ beliefs in their capacity to perform tasks influence their goals, persistence, and choices. In writing contexts, writing self-efficacy captures learners’ judgments about their ability to plan, monitor, revise, and complete written texts (Guo and Li, 2024; Payaprom and Payaprom, 2025). Self-efficacy is a key factor that drives human agency, referring to individuals’ beliefs in their ability to perform tasks, which in turn influences their goals, persistence, and choices. Those with strong self-efficacy are more likely to engage persistently and strategically in writing tasks. Conversely, individuals with weak self-efficacy may experience avoidance, negative self-talk, or anxiety, hindering their writing performance.

Contemporary empirical studies have continued to affirm the centrality of self-efficacy in writing. For example, Lin et al. (2024) found that writing self-efficacy among Chinese EFL learners was significantly associated with both self-regulated learning (SRL) writing strategies and writing achievement, confirming its direct and mediated effects in L2 writing contexts. Moreover, Jin et al. (2025) examined how learners’ use of generative AI tools in writing mediates self-efficacy and SRL strategy deployment, underscoring that even in technologically mediated tasks, confidence remains a potent predictor. In investigative contexts, learners with low self-efficacy often report greater writing anxiety, reduced strategic engagement, and poorer performance.

#### 2.3.2 Self-regulated learning (SRL) theory as mediator

SRL theory (Zimmerman, 2013; Zimmerman, 1990) describes how learners purposively manage their cognition, metacognition, behavior, and motivation to attain goals. In writing, SRL strategies include planning (e.g., outlining), monitoring (checking coherence, content flow), revising (local/global edits), and seeking feedback or peer input (Hwang, 2025). These strategies serve as the mechanistic bridge through which belief (self-efficacy) becomes regulated action, which then mitigates anxiety and enhances writing performance. Recent research continues to affirm the mediating power of SRL strategies. A meta-analysis by Guo and Bai (2022) in L2 learning contexts found that SRL interventions consistently had positive effects on language achievement and strategy use, and increased self-efficacy, though effect sizes varied across domains and measurement methods. In writing-specific contexts, Guo et al. (2021) demonstrated that SRL-based instruction led to substantial improvements in EFL students’ academic writing across varying levels of self-efficacy. This suggests that scaffolding SRL processes can help learners, even those with weaker self-beliefs, to perform better.

#### 2.3.3 Cognitive load theory (CLT) and the impact of anxiety on performance

CLT (Chandler and Sweller, 1991) delineates limits of working memory and posits that instructional or task demands beyond capacity hinder performance. CLT categorizes load into intrinsic load (task complexity), extraneous load (inefficiencies or distractions), and germane load (effort toward schema construction). Writing anxiety acts as a form of extraneous or interfering load: intrusive thoughts, worry, self-monitoring, and emotional arousal consume working memory resources, thereby reducing the capacity available for the core writing operations of idea generation, organization, linguistic encoding, and revision. Recent empirical work substantiates this mechanism in writing contexts. For instance, a study on L2 writers Wang et al. (2024a) modelled the relationships among cognitive load (subjective reports), anxiety, and writing performance and found that anxiety significantly predicted higher cognitive load, which in turn predicted lower performance. This provides direct empirical backing for the mediating role of cognitive load between affect (anxiety) and performance. Additionally, the same authors note that the load–anxiety–writing interplay is particularly pronounced in low-proficiency writers.

#### 2.3.4 Integrative framework: synthesizing SCT + SRL + CLT

Bringing SCT, SRL, and CLT into dialogue offers a theoretically rich and empirically grounded lens for understanding the framework:

From a theoretical standpoint, SCT conceptualizes writing self-efficacy as a motivational driver initiating strategic behavior and influencing persistence, with potential direct effects on performance and affect. Building on this motivational foundation, SRL theory specifies how learners convert confidence into regulated action by deploying cognitive, metacognitive, social, and motivational strategies. In parallel, CLT explains how emotional states, particularly anxiety, function as cognitive drains that may attenuate the effectiveness of strategy deployment by reducing the working memory resources available for executing writing processes.

Thus, the integrated model positions writing self-efficacy as an upstream belief variable, whose influence is mediated through an SRL-driven strategy system, but whose gains are moderated by cognitive load from anxiety (i.e., emotional interference). The model implies both sequential mediation (efficacy → strategies → anxiety → performance) and parallel paths (efficacy → performance, efficacy → anxiety). A confident writer with high self-efficacy is more likely to engage in planning, monitoring, revising, and seeking feedback as part of self-regulated learning strategies. The use of these strategies helps to structure the writing process, distribute cognitive effort efficiently, and mitigate frustration or doubt, thereby reducing writing-related anxiety and emotional strain. Lower anxiety, in turn, decreases the extraneous cognitive load imposed on working memory, freeing additional capacity for core writing operations and consequently enhancing performance. At the same time, self-efficacy may exert a direct positive influence on writing performance through increased persistence and resilience, as well as a direct negative influence on anxiety, since confident writers tend to anticipate fewer intrusive worries.

### 2.4 Current study

The integration of SCT, SRL, and CLT theories provides a comprehensive framework for understanding the dynamic relationships among writing self-efficacy, self-regulated writing strategies, writing anxiety, and writing performance. SCT emphasizes the central role of self-efficacy in motivating and guiding learning behaviors: students who are confident in their writing abilities are more likely to engage in cognitive, metacognitive, and social-behavioral strategies, thereby reducing anxiety and enhancing performance. SRL theory complements this view by explaining how these strategies act as self-initiated regulatory processes that translate motivational beliefs into effective task engagement. Learners who plan, monitor, and revise their work, or who seek feedback proactively, are typically better equipped to manage the emotional demands of writing. Finally, CLT highlights the detrimental impact of anxiety on performance, showing that excessive worry imposes extraneous cognitive load that interferes with idea generation, organization, and revision.

Building upon these theoretical perspectives, the present study employs a second-order SEM approach to examine how self-efficacy, strategy use, and anxiety interact to predict writing performance among EFL learners. Specifically, this study seeks to address the following focused research questions:

1. To what extent does writing self-efficacy predict the use of self-regulated writing strategies, and how do these strategies relate to students’ writing anxiety and performance?
2. Do different dimensions of self-regulated writing strategies (cognitive, metacognitive, motivational regulation, and social behavior) contribute uniquely to the reduction of writing anxiety and the enhancement of writing performance?
3. How do distinct components of writing anxiety (cognitive, somatic, and avoidance) differentially influence students’ writing performance?
4. Do self-regulated writing strategies and writing anxiety function as sequential mediators linking self-efficacy to writing performance within the proposed theoretical model?

**Figure 1:**
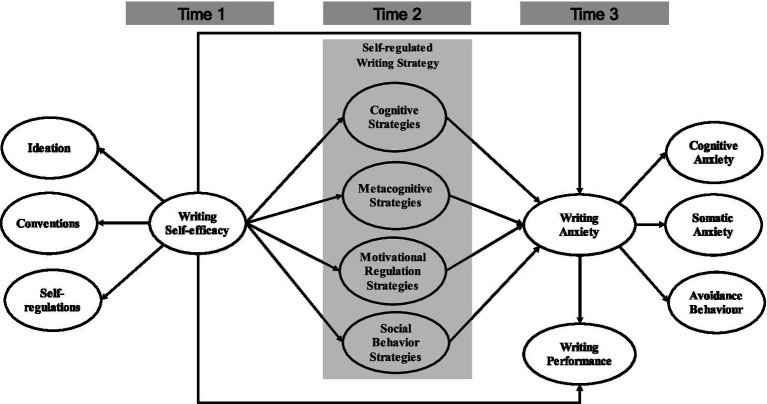
Research model.

By addressing these questions, the study aims to deepen understanding of how motivational beliefs and regulatory behaviors jointly shape writing outcomes in EFL contexts. The proposed framework was empirically tested using SEM, as illustrated in Figure 1, to evaluate the model’s overall fit and the magnitude of hypothesized associations among constructs.

## 3 Materials and methods

### 3.1 Sample size

**Figure 2:**
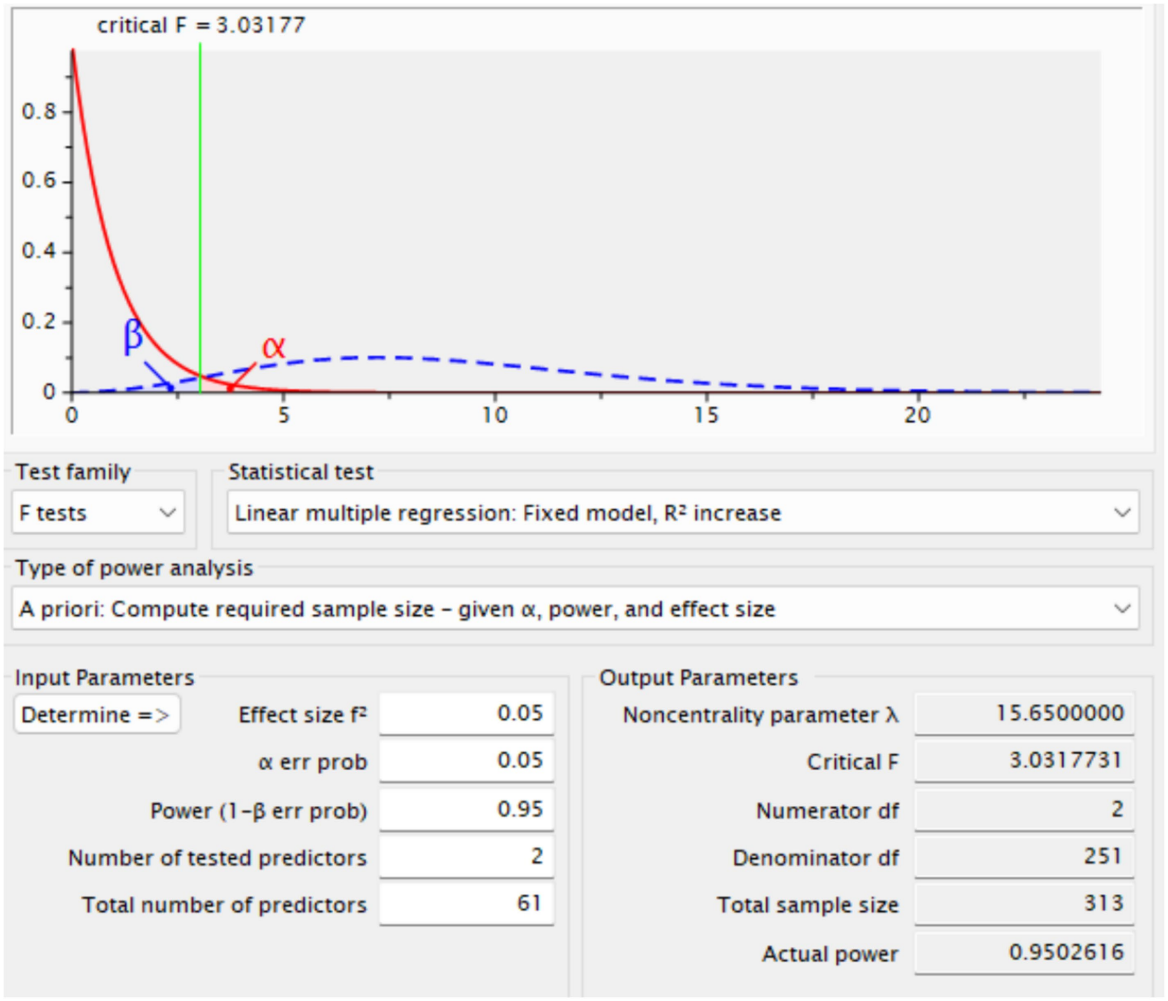
Sample size estimation.

A power analysis performed with G*Power software established that a minimum sample size of 311 participants was necessary to attain sufficient statistical power for this investigation (See Figure 2). The computation was predicated on an expected effect size of 0.05, a significance level (alpha) of 0.05, and a desired power of 0.95, so guaranteeing a substantial probability of identifying significant effects. The survey questions were initially formulated in English and thereafter meticulously evaluated by three bilingual specialists fluent in both Chinese and English. To guarantee translation precision, a back-translation technique was utilized, comprising an initial translation from English to Chinese followed by a retranslation into English. This procedure guaranteed uniformity and lucidity between the two linguistic variants. To recruit participants for this study, emails were sent to 20 English institutes across three provinces in China which are Anhui, Zhejiang, and Shandong requesting collaboration. Out of these, 12 institutes agreed to participate, allowing for a diverse yet regionally representative sample of EFL learners.

### 3.2 Measures

#### 3.2.1 Writing self-efficacy

This study assesses writing self-efficacy utilizing the framework established by Bruning et al. (2013), which defines writing self-efficacy as a multifaceted construct that includes several elements of the writing process. The study encompasses three principal dimensions: ideation (5 items), conventions (5 items), and self-regulations (6 items). Ideation evaluates students’ confidence in developing and structuring ideas for writing assignments, indicating their capacity to generate content proficiently. Conventions assess self-efficacy in the use of correct grammar, punctuation, and structural precision, reflecting confidence in mastering writing mechanics. Self-regulations assess students’ assessed competence in planning, monitoring, and revising their work, highlighting their potential for self-directed enhancement. By incorporating these characteristics, the study delivers a thorough evaluation of students’ confidence in their writing skills, elucidating how various facets of self-efficacy influence writing performance and anxiety regulation in EFL learners.

#### 3.2.2 Self-regulated writing strategy

Self-regulated writing strategy was measured using the framework proposed by Teng and Zhang (2016), which classifies writing strategies into four principal dimensions: Cognitive Strategies (8 items), Metacognitive Strategies (8 items), Social Behavior Strategies (8 items), and Motivational Regulation Strategies (8 items). Cognitive techniques emphasize the pragmatic elements of writing, including the organization of ideas, content structuring, and effective draft revision. Metacognitive techniques analyze students’ capacity to organize, monitor, and assess their writing development, thereby facilitating a systematic and objective-driven methodology for writing assignments. Social behavior strategies assess the degree to which students solicit feedback, collaborate with others, and participate in social interactions to improve their writing abilities. Motivational regulation strategies assess how students maintain their motivation and perseverance when confronted with writing difficulties, encompassing techniques to retain engagement and surmount problems.

#### 3.2.3 Writing anxiety

Writing anxiety was operationalized based on the model developed by Cheng (2004), which defines writing anxiety as a multidimensional construct comprising Cognitive Anxiety (9 items), Somatic Anxiety (9 items), and Avoidance Behavior (9 items). Cognitive anxiety pertains to the psychological distress linked to writing assignments, characterized by excessive concern, apprehension of negative assessment, and self-doubt regarding writing proficiency. Somatic anxiety encompasses the physiological manifestations of writing-related stress, such as uneasiness, elevated heart rate, and physical discomfort during writing tasks. Avoidance behavior denotes pupils’ propensity to postpone, retreat from writing assignments, or entirely evade circumstances necessitating written expression owing to apprehension and worry. This study offers a thorough grasp of the effects of writing anxiety on students’ writing experiences, facilitating a more profound investigation of how various types of anxiety affect writing performance and method utilization in EFL learners.

#### 3.2.4 Writing performance

The writing performance of students in this study was evaluated by an argumentative essay on a topic chosen from the Test of English Majors—Band 4 (TEM4), a standardized national examination aimed at assessing the English ability of English-major students in China. The TEM thoroughly evaluates students’ language competencies, encompassing listening, speaking, reading, writing, vocabulary, and translation, thereby serving as a dependable instrument for gauging academic English proficiency. This study employed global scoring as the preferred evaluation method according to the official grading scheme, whereby two independent raters assigned scores based on their overall impression of the essay’s quality, rather than concentrating on specific grammatical or structural errors. The scoring system included five predetermined score levels (2, 5, 8, 11, and 14 out of 15), accompanied by comprehensive descriptors to delineate various degrees of writing performance. A score of 2 signifies essays that lack logical coherence, demonstrate fragmented language, and include significant grammatical errors, reflecting inadequate writing proficiency. A score of 14 indicates essays that exhibit substantial relevance to the topic, articulate and coherent expression of thinking, and logical argumentation with few or no significant language errors, signifying elevated writing proficiency. This holistic grading approach guarantees that students’ writing is evaluated comprehensively, considering both content quality and language proficiency, rather than penalizing insignificant errors that do not substantially affect meaning. Utilizing this strategy ensures the scoring procedure upholds consistency and equity, while concurrently conforming to established assessment protocols in academic writing evaluations. Table 1 presents research variable measurement results.

**Table 1:** Research variables’ measurement.

Construct	Sub-dimensions	Number of items	Source
Writing self-efficacy	Ideation	5	Bruning et al. (2013)
	Conventions	5	
	Self-regulations	6	
Self-regulated writing strategies	Cognitive strategies	8	Teng and Zhang (2016)
	Metacognitive strategies	8	
	Motivational regulation strategies	8	
	Social behavior strategies	8	
Writing anxiety	Cognitive anxiety	9	Cheng (2004)
	Somatic anxiety	9	
	Avoidance behavior	9	
Writing performance	Argumentative essay (TEM4-based)	1 (task)	TEM4 scoring scheme

The questionnaire instruments developed in English were translated into Chinese following a standard translation and back-translation procedure to ensure linguistic accuracy and conceptual equivalence. First, two bilingual researchers independently translated the original English items into Chinese. Subsequently, a separate bilingual expert, who was blind to the original versions, back-translated the Chinese items into English. The back-translated versions were then systematically compared with the original English instruments, and any discrepancies were discussed and resolved through consensus among the research team. This process ensured that the final Chinese versions accurately captured the intended meanings of the original constructs and were appropriate for the Chinese EFL context.

### 3.3 Data collection procedure and participant characteristics

We implemented a three-wave longitudinal design to examine whether the associations specified in the theoretical framework were temporally ordered and statistically consistent with the proposed sequential pathways—writing self-efficacy → self-regulated writing strategies → writing anxiety → writing performance. Similar three-wave longitudinal designs have been successfully employed in prior studies to examine temporally ordered relationships among motivational, cognitive, and affective variables (Abdelmoteleb, 2019; Yuan et al., 2021). Temporal separation between measurements helped reduce same-time common-method variance and permitted testing lagged and cross-lagged relations consistent with the underlying theories, while carefully avoiding causal over interpretation. The surveys were administered approximately every six weeks (T1 → T2 → T3; total ≈ 12 weeks), aligning with a typical university semester that includes two formative feedback cycles before the final writing assessment. This spacing provided sufficient time for observable changes in strategy use and affective states to emerge. The principal analytic focus was on model-consistent sequential (lagged) indirect effects, specifically T1 self-efficacy → T2 self-regulated writing strategies → T3 anxiety and performance, controlling for prior levels of anxiety and relevant covariates.

All three survey waves were distributed through WeChat, a widely used communication and learning platform among university students in China. Official WeChat class groups served as the distribution channels to ensure standardized access and timing. Participants received a secure survey link, along with a brief explanation of the study’s purpose and assurances of confidentiality. Using WeChat provided a familiar and easily navigable interface, which enhanced accessibility, reduced technical barriers, and encouraged consistent participation throughout the longitudinal process. To ensure reliable data linkage across waves, each participant was assigned a unique, anonymous identification code that they entered at each data-collection point. This procedure preserved anonymity while allowing the researchers to track matched responses over time.

Across the three waves, a total of 468 complete responses were obtained, yielding an overall response rate of 93.6%. This strong participation rate ensured that the study met its intended statistical power requirements and maintained data quality throughout the longitudinal sequence. The final sample included 215 male and 253 female English-as-a-Foreign-Language (EFL) learners, all of whom were native Mandarin speakers enrolled in academic English courses at several comprehensive universities in eastern China. Participants’ ages ranged from 25 to 30 years (Mean = 27.15, SD = 1.32). This sample can be considered broadly representative of university-level EFL learners in eastern China, a population for whom academic writing constitutes a core component of English instruction and assessment. The participants shared a relatively homogeneous linguistic, cultural, and educational background, which minimized extraneous variance stemming from regional or dialectal differences. This homogeneity, coupled with the high completion rate, contributed to both measurement reliability and linguistic precision in interpreting self-report instruments across waves.

Together, the use of a multi-wave design, a familiar digital platform, and an engaged and homogeneous participant group ensured the collection of high-quality longitudinal data suitable for advanced structural modelling of writing self-efficacy, strategy use, anxiety, and performance in an authentic EFL learning environment.

## 4 Results

### 4.1 Descriptive statistics of research variables

Table 2 presents the descriptive statistics for all primary study variables, including writing self-efficacy, self-regulated writing strategies (cognitive, metacognitive, motivational regulation, and social behavior), writing anxiety (cognitive, somatic, and avoidance), and writing performance. The descriptive analysis indicates moderate to high mean levels of writing self-efficacy and strategy use, suggesting that participants generally perceived themselves as capable writers who actively employed regulatory strategies during writing tasks. Writing anxiety dimensions displayed moderate mean scores, indicating that although students experienced some level of apprehension, anxiety did not reach debilitating levels. Notably, somatic anxiety reported the highest mean among the three anxiety types, consistent with prior studies indicating that physiological symptoms such as tension or heart rate are the most common stress manifestations among EFL writers. Writing performance showed a moderate to high mean level, with acceptable variability, indicating sufficient dispersion for subsequent structural modelling. The skewness and kurtosis values for all variables fell within ±1, suggesting approximate normality and suitability for parametric analyses. Overall, the descriptive patterns align with theoretical expectations: higher writing self-efficacy and greater use of self-regulated strategies are associated with lower anxiety and better writing outcomes among EFL learners.

**Table 2:** Descriptive statistics.

Variable	Mean (M)	Standard deviation (SD)	Skewness	Kurtosis	Range (observed)	Cronbach’s *α*
Writing self-efficacy	4.12	0.67	−0.43	−0.12	1.8–5.0	0.93
Self-regulated writing strategy	4.05	0.58	−0.37	−0.21	2.1–5.0	0.94
Cognitive strategies	4.09	0.63	−0.41	0.06	1.9–5.0	0.88
Metacognitive strategies	4.15	0.59	−0.35	−0.17	2.0–5.0	0.91
Motivational regulation strategies	3.84	0.71	−0.22	−0.45	1.6–5.0	0.87
Social behavior strategies	3.96	0.68	−0.29	−0.23	1.7–5.0	0.89
Writing anxiety	3.05	0.74	0.26	−0.34	1.2–4.9	0.92
Cognitive anxiety	3.08	0.77	0.22	−0.48	1.0–5.0	0.88
Somatic anxiety	3.23	0.79	0.18	−0.57	1.1–5.0	0.9
Avoidance behavior	2.85	0.83	0.31	−0.39	1.0–5.0	0.86

### 4.2 Common method bias

Common Method Bias (CMB) refers to spurious covariance shared among variables due to the measurement method rather than the constructs themselves (Podsakoff et al., 2003; Podsakoff et al., 2012). Although the study employed validated self-report instruments for all psychological constructs, the use of a single data source raises the potential for CMB, which may inflate observed correlations among variables due to shared measurement context rather than true underlying relationships. To minimize such bias, several procedural and statistical strategies were applied. Procedurally, constructs were measured across three distinct time points (T1–T3), thereby introducing temporal separation that reduces same-time response consistency. Participants were assured of anonymity and informed that there were no right or wrong answers to decrease social desirability pressure. In addition, item order randomization was used within each questionnaire to minimize response patterning. Statistically, the dataset was examined for potential method effects through Harman’s single-factor test, which indicated that no single factor accounted for most of the covariance among items, suggesting that CMB was not a dominant concern. In line with Podsakoff et al. (2003, 2012), this statistical test was used as a supplementary diagnostic tool in combination with procedural remedies implemented at the design stage. The full collinearity assessment approach proposed by Kock (2015) was not adopted, as it is most commonly applied and validated in variance-based PLS-SEM models. Nevertheless, given the reliance on self-report measures, residual bias cannot be entirely ruled out, and future research is encouraged to adopt multi-method or multi-source designs—for example, triangulating self-report data with behavioral indicators (e.g., keystroke logging, feedback-request counts) or teacher evaluations—to further validate the observed relationships.

### 4.3 Reliability of the measures

To ensure the internal consistency and reliability of the instruments used in this study, Cronbach’s alpha (*α*) and Composite Reliability (CR) values were computed for each construct and its respective sub dimensions. All constructs exhibited high reliability coefficients, exceeding the recommended threshold of 0.70 (Nunnally and Bernstein, 1994), indicating strong internal consistency across items. Specifically, writing self-efficacy and self-regulated writing strategies demonstrated excellent reliability (*α* = 0.93 and 0.94, respectively), reflecting the stable measurement of motivational and strategic behaviors. The sub dimensions of self-regulated strategies, including cognitive, metacognitive, motivational regulation, and social-behavioral components, also displayed strong reliability values (α ranging from 0.87 to 0.91).

Similarly, the three subcomponents of writing anxiety (cognitive, somatic, and avoidance) yielded Cronbach’s alpha values between 0.86 and 0.90, supporting their reliability as distinct yet interrelated affective factors. Writing performance, rated independently by two trained raters using a standardized rubric, achieved an inter-rater reliability (ICC) of 0.91, confirming high agreement and consistency between raters. The composite reliability indices (CR = 0.88 to 0.95) further confirmed the convergent consistency of each latent construct. Together, these results indicate that all psychometric measures employed in this study possess robust internal consistency and are suitable for inclusion in subsequent structural modeling analyses.

### 4.4 SEM outputs

The results in Table 3 demonstrate the important role of writing self-efficacy in influencing self-regulated writing strategies, writing anxiety, and writing performance, with notable gender differences. For both females and males, writing self-efficacy positively predicts the use of cognitive, metacognitive, and social behavior strategies, but the effect sizes are notably higher for males (0.784 for cognitive strategy, 0.778 for metacognitive strategy, and 0.638 for social behavior strategy) compared to females (0.521, 0.576, and 0.386, respectively). This suggests that males with higher writing self-efficacy tend to employ writing strategies more frequently than females. Additionally, writing self-efficacy has a strong negative effect on writing anxiety (−0.645 for females and −0.603 for males), indicating that students who are more confident in their writing abilities experience lower levels of writing anxiety. The positive impact of writing self-efficacy on writing performance is stronger for females (0.621) than for males (0.558), suggesting that female students’ writing performance is more directly influenced by their self-confidence, whereas for males, strategy use may play a more indirect role in determining performance.

**Table 3:** Direct and indirect effects.

Effects	Standardized coefficient	S. E.	*p*-value
Females			
Direct effect			
Writing self-efficacy (T1) → cognitive strategies (T2)	0.521	0.06	< 0.001
Writing self-efficacy (T1) → metacognitive strategies (T2)	0.576	0.06	< 0.001
Writing self-efficacy (T1) → social behavior strategies (T2)	0.386	0.07	< 0.001
Writing self-efficacy (T1) → motivational regulation strategies (T2)	0.087	0.10	0.382
Writing self-efficacy (T1) → writing anxiety (T3)	−0.645	0.07	< 0.001
Writing self-efficacy (T1) → writing performance (T3)	0.621	0.06	< 0.001
Cognitive strategies (T2) → writing anxiety (T3)	−0.457	0.07	< 0.001
Metacognitive strategies (T2) → writing anxiety (T3)	−0.687	0.06	< 0.001
Social behavior strategies (T2) → writing anxiety (T3)	−0.403	0.07	< 0.001
Writing anxiety (T3) → writing performance (T3)	−0.532	0.06	< 0.001
Indirect effect			
Writing self-efficacy (T1) → cognitive strategies (T2) → writing anxiety (T3)	−0.238	0.05	< 0.001
Writing self-efficacy (T1) → metacognitive strategies (T2) → writing anxiety (T3)	−0.396	0.06	< 0.001
Writing self-efficacy (T1) → social behavior strategies (T2) → writing anxiety (T3)	−0.156	0.05	0.002
Males			
Direct effect			
Writing self-efficacy (T1) → cognitive strategies (T2)	0.784	0.05	< 0.001
Writing self-efficacy (T1) → metacognitive strategies (T2)	0.778	0.05	< 0.001
Writing self-efficacy (T1) → social behavior strategies (T2)	0.638	0.06	< 0.001
Writing self-efficacy (T1) → motivational regulation strategies (T2)	0.109	0.09	0.231
Writing self-efficacy (T1) → writing anxiety (T3)	−0.603	0.07	< 0.001
Writing self-efficacy (T1) → writing performance (T3)	0.558	0.06	< 0.001
Cognitive strategies (T2) → writing anxiety (T3)	−0.288	0.08	< 0.001
Metacognitive strategies (T2) → writing anxiety (T3)	−0.619	0.06	< 0.001
Social behavior strategies (T2) → writing anxiety (T3)	−0.655	0.06	< 0.001
Writing anxiety (T3) → writing performance (T3)	−0.547	0.06	< 0.001
Indirect effect			
Writing self-efficacy (T1) → cognitive strategies (T2) → writing anxiety (T3)	−0.226	0.06	< 0.001
Writing self-efficacy (T1) → metacognitive strategies (T2) → writing anxiety (T3)	−0.482	0.07	< 0.001
Writing self-efficacy (T1) → social behavior strategies (T2) → writing anxiety (T3)	−0.418	0.06	<0.001

The analysis revealed that among all correlations, the impact of writing self-Efficacy on motivational regulation strategies was not significant for both male and female participants. This suggests that writing self-efficacy does not directly predict students’ ability to regulate their motivation during the writing process. Unlike cognitive, metacognitive, and social strategies, which showed significant relationships with writing self-efficacy, motivational regulation strategies did not exhibit a meaningful effect, indicating that students’ confidence in their writing abilities does not necessarily translate into their ability to sustain motivation and persistence in writing tasks.

Furthermore, the relationship between writing strategies and writing anxiety highlights some key gender differences. Metacognitive strategies have the strongest negative impact on writing anxiety for both groups (−0.687 for females and −0.619 for males), indicating that students who engage in planning, goal-setting, and self-monitoring experience reduced anxiety. However, males appear to rely more on social behavior strategies (−0.655) to mitigate anxiety compared to females (−0.403), suggesting that male students may benefit more from external feedback and collaboration to manage stress. The indirect effects further confirm the mediating role of writing strategies in reducing anxiety, particularly through metacognitive strategies, which show the strongest indirect effect (−0.396 for females and −0.482 for males). This suggests that while self-efficacy directly lowers anxiety, its impact is further enhanced through effective strategy use. Overall, these findings emphasize the importance of promoting self-regulated writing strategies tailored to gender-specific needs to improve writing outcomes and reduce anxiety in EFL learners.

### 4.5 Multi group analysis

The multi group analysis results in Table 4 examine the relationships between writing self-efficacy, self-regulated writing strategies (cognitive, metacognitive, and social), writing anxiety, and writing performance. The direct effects show that writing self-efficacy significantly predicts the use of cognitive (*χ*^2^ = 4.93, *p* < 0.001), metacognitive (*χ*^2^ = 3.67, *p* < 0.001), and social strategies (*χ*^2^ = 4.11, *p* < 0.001), indicating that students with higher writing self-efficacy are more likely to employ these strategies to regulate their writing process. However, writing self-efficacy does not significantly predict motivational regulation strategies (*χ*^2^ = 0.65, *p* = 0.476) or writing anxiety (*χ*^2^ = 0.98, *p* = 0.378), suggesting that self-efficacy alone is not a direct determinant of anxiety but may exert influence through other factors such as strategy use. In contrast, writing self-efficacy shows a significant direct association with writing performance (*χ*^2^ = 2.66, *p* = 0.045).

**Table 4:** Multi group analysis.

Relationships	$\text{Wald}\;\chi^{2}$ test	*p*-value
*Direct effects*		
Writing self-efficacy (T1) → cognitive strategies (T2)	4.93	<0.001
Writing self-efficacy (T1) → metacognitive strategies (T2)	3.67	<0.001
Writing self-efficacy (T1) → social behavior strategies (T2)	4.11	<0.001
Writing self-efficacy (T1) → motivational regulation strategies (T2)	0.65	0.476
Writing self-efficacy (T1) → writing anxiety (T3)	0.98	0.378
Writing self-efficacy (T1) → writing performance (T3)	2.66	0.045
Cognitive strategies (T2) → writing anxiety (T3)	1.09	0.245
Metacognitive strategies (T2) → writing anxiety (T3)	4.36	<0.001
Social behavior strategies (T2) → writing anxiety (T3)	2.58	0.031
*Indirect effects*		
Writing self-efficacy (T1) → cognitive strategies (T2) → writing anxiety (T3)	0.24	0.672
Writing self-efficacy (T1) → metacognitive strategies (T2) → writing anxiety (T3)	1.17	0.098
Writing self-efficacy (T1) → social behavior strategies (T2) → writing anxiety (T3)	4.78	<0.001

Additionally, regarding the effects of writing strategies on writing anxiety, the path from cognitive strategy to writing anxiety does not differ significantly across gender (*χ*^2^ = 1.09, *p* = 0.245). However, significant gender differences were identified for the paths from metacognitive strategy to writing anxiety (*χ*^2^ = 4.36, *p* < 0.001) and from social behavior strategy to writing anxiety (*χ*^2^ = 2.58, *p* = 0.031), suggesting that the influence of these strategies on anxiety varies between male and female students.

The indirect effects provide further insights into how writing self-efficacy influences writing anxiety through different writing strategies. Notably, the mediation effect of cognitive strategies on the relationship between writing self-efficacy and writing anxiety is not significant (*χ*^2^ = 0.24, *p* = 0.672), suggesting that simply using structured cognitive strategies may not be sufficient to reduce anxiety. Similarly, the indirect effect of metacognitive strategies becomes marginal in the multi group model (*χ*^2^ = 1.17, *p* = 0.098), suggesting that although metacognitive strategies represent the strongest mediator in the overall structural model, their mediating role is not equally robust across gender groups. However, the indirect effect of social strategies on writing anxiety is highly significant (*χ*^2^ = 4.78, *p* < 0.001), suggesting that students with higher writing self-efficacy who actively seek social support experience lower anxiety levels. This highlights the crucial role of social interactions, such as peer feedback and collaborative learning, in mitigating writing-related stress. These findings suggest that while self-efficacy enhances the use of various writing strategies, its anxiety-reducing effects are most evident when students engage in social strategies. Therefore, interventions aimed at reducing writing anxiety should emphasize peer support and collaborative writing practices rather than relying solely on individual cognitive or metacognitive regulation.

## 5 Discussion and conclusion

### 5.1 Discussion

The primary aim of this study is to illustrate how bolstering writing self-efficacy and advocating for self-regulated writing strategy might alleviate writing anxiety and enhance writing performance, especially in EFL situations.

First, this study contributes to studies on writing performance by revealing strong negative connections between self-regulated writing strategies (cognitive, metacognitive, and social behavior strategies) and writing anxiety among EFL learners of both genders. Specifically, SEM results (Table 3) indicate that cognitive (*β* = −0.457 for females; *β* = −0.288 for males), metacognitive (*β* = −0.687 for females; *β* = −0.619 for males), and social behavior strategies (*β* = −0.403 for females; *β* = −0.655 for males) all exert statistically significant negative effects on writing anxiety. The results indicate that students who employ self-regulated writing procedures generally exhibit reduced writing anxiety, a key determinant of their overall writing achievement. Cognitive strategies, including content organization, paragraph structuring, and revision approaches, enable learners to methodically tackle writing problems, thereby diminishing doubt and bolstering their confidence (Gao, 2023). Likewise, metacognitive strategies, encompassing planning, goal-setting, self-monitoring, and reflection empower learners to manage their writing process, thus alleviating worry and tension (Blasco, 2016). Social behavior strategies, including soliciting feedback from educators or peers, participating in discussions, and collaborating on writing assignments, mitigate writing anxiety by cultivating a sense of support and collective learning experiences (Xiong et al., 2024). These findings corroborate other research, including that of Hwang (2025) as well as Sari and Han (2024), which demonstrated that self-regulated learning strategies markedly alleviated anxiety and enhanced writing performance among EFL students. Likewise, Yeung et al. (2024) emphasized that self-regulated learners demonstrated reduced writing anxiety and increased writing motivation, supporting the assertion that strategic writing behaviors can mitigate psychological obstacles and improve academic performance. These findings, along with the current research, furnish persuasive evidence that fostering SRL in writing instruction can assist students in alleviating anxiety and enhancing their confidence in writing skills.

Moreover, the gender-neutral impact of self-regulated writing strategies on writing anxiety offers valuable insight into the applicability of these strategies among various learner demographics (Verma and Gupta, 2024). This study reveals that both males and females significantly benefit from self-regulated strategies in writing, despite employing slightly different mechanisms, contrary to earlier studies suggesting that gender differences may affect writing regulation and anxiety management (Schuetze and Yan, 2024). Males seemed to depend more on social behavior strategies, including peer feedback and collaborative writing, to alleviate writing anxiety, while females shown a greater inclination towards metacognitive strategies, such as planning and self-monitoring, to address their writing difficulties. The findings align with research by Zhu et al. (2024), which indicated that female students are more inclined towards reflective and goal-setting activities, whereas male students derive greater advantages from interactive and socially oriented learning experiences. Notwithstanding these nuanced distinctions, the prevailing trend substantiates that self-regulation is a potent strategy for alleviating writing anxiety in both genders, underscoring the necessity for instructional methodologies that integrate self-regulated learning techniques as fundamental elements of EFL writing pedagogy. Educators should prioritize the integration of structured self-regulated writing programs that address both cognitive and emotional dimensions of writing. Instructors can assist students in developing resilience to writing anxiety, cultivating independent learning habits, and eventually improving their writing ability in both academic and real-world contexts.

Second, the results of this study indicate a strong negative correlation between writing anxiety and writing performance, with SEM outputs confirming significant negative effects for both females (*β* = −0.532, *p* < 0.001) and males (*β* = −0.547, *p* < 0.001), as reported in Table 3. The larger absolute coefficient for female learners suggests that writing anxiety exerts a stronger performance-impairing effect among females than among males. This discrepancy may be due to gender-based differences in coping mechanisms, where female students might be more affected by emotional and physiological stressors during writing. The hierarchical structure of writing anxiety analyzed in this study provides a deeper understanding of its varying influences on writing performance, making this the first study to apply a second-order structural equation modelling approach to explore these relationships comprehensively. Previous studies have generally treated writing anxiety as a single construct rather than analyzing how each dimension independently contributes to writing difficulties.

The results align with past research that emphasizes the detrimental effects of anxiety on writing performance but diverge by offering a more nuanced, multi-layered interpretation. For instance, Fan and Wang (2024) identified writing anxiety as a major factor affecting learners’ writing quality but did not differentiate between cognitive, somatic, and behavioral components. Razmi and Ghane (2024) found that writing self-efficacy played a crucial role in mitigating anxiety, but their research lacked a detailed breakdown of how different anxiety types interact with performance outcomes. Abdi Tabari and Goetze (2024) also explored anxiety’s effects on second-language acquisition, concluding that physiological symptoms of anxiety hinder fluency and cognitive processing, which supports this study’s finding that somatic anxiety has the most substantial impact on writing performance. Another relevant study by Sari and Han (2024) examined the effects of foreign language anxiety on writing but did not explore avoidance behavior as a distinct factor, which this study found to be the second most influential predictor of lower writing performance. The inclusion of avoidance behavior as a separate component provides a novel perspective on how students’ reluctance to engage in writing directly affects their success (Aydın and Yüce, 2024). Given these findings, educators and researchers should focus on developing targeted interventions to reduce somatic anxiety, address avoidance tendencies, and equip students with cognitive strategies to manage writing-related stress. Future studies should expand on this model by examining long-term trends in writing anxiety’s impact on performance and exploring whether different instructional approaches can effectively mitigate its negative effects across diverse learner populations.

Third, this study contributes to the understanding of the mediating role of self-regulated writing strategies in the relationship between writing self-efficacy and writing anxiety, highlighting how students with higher writing self-efficacy are more likely to adopt cognitive, metacognitive, and social strategies, which in turn help reduce their anxiety levels. The results show that metacognitive strategies have the strongest mediating effect on writing anxiety for both females (−0.396) and males (−0.482), suggesting that students who engage in planning, self-monitoring, and reflective evaluation experience lower anxiety when writing. Cognitive strategies also contribute to anxiety reduction (−0.238 for females and −0.226 for males), but their effect is not as strong as metacognitive strategies, implying that while structuring ideas and organizing content is helpful, the ability to regulate and reflect on one’s writing process plays a more substantial role in lowering anxiety. Additionally, social behavior strategies mediate writing anxiety reduction (−0.156 for females and −0.419 for males), with a notably stronger effect among males, indicating that male students benefit more from external feedback and peer collaboration to manage their writing-related stress. The direct effect of writing self-efficacy on writing anxiety (−0.645 for females and −0.603 for males) further confirms that students who believe in their writing abilities experience less anxiety, but this relationship is reinforced when self-regulated writing strategies are actively employed. These findings suggest that enhancing self-efficacy alone is not enough to lower writing anxiety; students must also develop strategic learning behaviors to effectively regulate their writing processes. By emphasizing the development of self-regulated learning strategies, particularly metacognitive and social behavior strategies, educators can provide students with the tools they need to alleviate writing anxiety and build confidence in their writing abilities.

Furthermore, this study examines the mediating influence of writing anxiety on the relationship between self-regulated writing strategies and writing performance, demonstrating that anxiety serves as a barrier that weakens the positive impact of strategic writing behaviors on writing performance. The results indicate that writing anxiety negatively affects writing performance for both females (−0.532) and males (−0.547), with a stronger detrimental effect on female students. This suggests that even if females employ effective self-regulated strategies, heightened anxiety can still hinder their writing outcomes. Among the different strategies, metacognitive strategies appear to have the greatest potential in offsetting anxiety’s negative impact on performance, which aligns with prior research suggesting that learners who actively plan and reflect on their writing demonstrate greater resilience against anxiety-driven performance decline. For males, social behavior strategies play a crucial role in moderating anxiety’s effects on writing performance, reinforcing the idea that peer support and external guidance can help mitigate the disruptive influence of anxiety. These findings emphasize the importance of reducing writing anxiety as a means of fully leveraging the benefits of self-regulated writing strategies on performance. Thus, educational interventions should not only promote SRL but also incorporate structured anxiety-reduction techniques, such as mindfulness exercises, writing workshops, and constructive feedback mechanisms, to help students manage anxiety and optimize their writing performance. This study’s second-order SEM approach provides an in-depth analysis of these complex relationships, offering valuable insights into how psychological and strategic factors interact to shape writing success.

### 5.2 Implications

This study’s findings provide substantial insights into the interconnection of writing self-efficacy, self-regulated writing techniques, writing anxiety, and writing performance among EFL learners. This research enhances both theoretical and empirical insights into the interaction of psychological and strategic elements affecting writing outcomes by integrating SCT, SRL, and CLT. The study’s findings underscore the mediating function of self-regulated writing methods in alleviating anxiety and enhancing writing performance, accentuating the significance of cognitive, metacognitive, and social behavior strategies in academic writing. Furthermore, the examination of various facets of writing anxiety (cognitive, somatic, and avoidance behavior) offers a more intricate comprehension of the differential impact of anxiety on writing performance among gender groups. These findings include significant significance for educators, curriculum developers, and researchers, providing direction on how to improve writing instruction, alleviate writing anxiety, and promote self-regulated learning habits. The subsequent sections examine the theoretical and empirical implications of the work, acknowledge its limits, and offer recommendations for future research to enhance its contributions.

#### 5.2.1 Theoretical implications

First, consistent with the observed direct effect of writing self-efficacy on writing anxiety (*β* = −0.645 for females; *β* = −0.603 for males), the findings extend Bandura (1986) by illustrating that writing self-efficacy significantly influences students’ writing behaviors, mitigates anxiety, and improves performance.

Second, the mediating roles of cognitive, metacognitive, and social behavior strategies provide empirical support for Zimmerman (1990) self-regulated learning model in the domain of L2 writing. Specifically, the stronger mediating effect of metacognitive strategies (*β* = −0.396 for females; *β* = −0.482 for males) indicates that planning, monitoring, and reflection constitute the core regulatory mechanisms through which self-efficacy translates into reduced writing anxiety.

Third, although cognitive load was not directly measured, the strong negative effects of writing anxiety on writing performance (*β* = −0.532 for females; *β* = −0.547 for males) provide indirect empirical support for Chandler and Sweller’s (1991) Cognitive Load Theory, suggesting that heightened anxiety may constrain learners’ available cognitive resources during writing tasks.

Fourth, the second-order structural equation model conceptualizes writing anxiety as a hierarchical construct and empirically demonstrates that its sub dimensions (somatic, avoidance, and cognitive anxiety) exert differential effects on writing performance, thereby advancing anxiety theory from a unitary to a multidimensional affective framework in L2 writing research.

Finally, the significant negative paths from self-regulated writing strategies to writing anxiety across all three strategy types indicate that strategic regulation functions as a key psychological mechanism for anxiety reduction, thereby extending existing SRL by explicitly integrating affective regulation into the writing process.

#### 5.2.2 Empirical implications

Based on the significant negative paths from all three self-regulated writing strategies to writing anxiety observed in this study, the findings indicate that students who actively employ cognitive, metacognitive, and social behavior strategies exhibit reduced writing anxiety, underscoring the necessity for explicit instruction in strategic writing behaviors.

Among these strategies, metacognitive strategies, such as planning, self-monitoring, and reflection, demonstrated the strongest anxiety-reducing effects, suggesting that fostering students’ self-regulation skills should be a primary instructional focus in EFL writing education.

The results further reveal gender-differentiated mediation patterns, with social behavior strategies exerting a stronger anxiety-reducing effect for males, while metacognitive strategies play a more prominent role for females. This suggests that writing instruction may benefit from flexible and differentiated pedagogical designs that accommodate learners’ diverse regulatory preferences.

In addition, the strong negative effects of writing anxiety on writing performance highlight the necessity for educators to integrate structured anxiety-reduction practices, such as guided feedback, scaffolder writing tasks, and confidence-building activities, into regular classroom instruction.

The important role of social behavior strategies in reducing anxiety, particularly for male students, further implies that peer feedback, collaborative writing, and interactive learning environments can serve as effective pedagogical tools for alleviating affective barriers in writing.

Finally, given the robust interconnections among writing self-efficacy, strategic regulation, and writing performance identified in this study, educational institutions are encouraged to develop integrated writing support programs that combine self-regulated learning training with systematic emotional support mechanisms.

### 5.3 Limitations and future directions

Despite the study’s methodological rigor and firm theoretical grounding, several limitations warrant consideration. First, although the design incorporated three measurement waves to enhance temporal separation and reduce same-time common-method variance, the analyses remain non-experimental. Structural equation modeling allows for testing model consistency with theory but cannot establish causal direction. Therefore, the reported relationships should be interpreted as statistical associations consistent with theoretical expectations, not definitive causal effects. Future research should employ cross-lagged panel, experimental, or intervention-based designs to examine whether changes in self-efficacy and strategy use indeed lead to subsequent reductions in anxiety and improvements in writing performance.

In addition, not all paths in the structural model were empirically supported. For example, writing self-efficacy did not show a consistent direct association with later writing anxiety or writing performance in the multi group model, and the path from self-efficacy to motivational regulation strategies was non-significant. Moreover, some indirect effects (e.g., via cognitive and metacognitive strategies) were non-significant or only marginally significant, suggesting that the strength and form of the belief–strategy–affect linkages may be conditional on specific strategy dimensions, analytic specifications, or subgroup characteristics. Therefore, conclusions should emphasize the supported pathways while treating unsupported links as hypotheses to be re-tested in future studies.

Moreover, the study relied exclusively on self-report instruments for measuring psychological constructs such as writing self-efficacy, self-regulated writing strategies, and writing anxiety. While the scales used were well-validated and demonstrated strong psychometric properties, self-reported data are inherently vulnerable to Common Method Bias (CMB), social desirability, and inaccuracies in self-perception. Several procedural remedies were implemented, such as anonymity assurances, randomized item ordering, and multi-wave administration, but residual bias cannot be entirely excluded. Future studies could enhance validity by incorporating multi-method or multi-source assessments, including teacher evaluations, peer ratings, or behavioral indicators such as keystroke dynamics, writing logs, or feedback-seeking frequencies, to capture both perceived and enacted self-regulation processes.

Third, the writing performance measure was limited to a single standardized argumentative essay evaluated by independent raters. While rubric-based scoring ensured inter-rater reliability and objectivity, a single essay cannot fully capture the multidimensional nature of writing competence, which spans multiple genres, rhetorical purposes, and cognitive demands. Consequently, caution should be exercised when generalizing the findings to broader writing proficiency. Future research would benefit from multi-task performance assessment (e.g., narrative, expository, and reflective writing), portfolio evaluations, or longitudinal writing development tracking to represent a more comprehensive view of students’ written communication skills.

Fourth, the participant pool consisted exclusively of Mandarin-speaking EFL learners enrolled in similar academic contexts. This linguistic and cultural homogeneity minimized extraneous variability and improved measurement precision. Still, it also limited the generalizability of the results to learners with different first languages, cultural backgrounds, or educational systems. Replicating the study across diverse populations, such as multilingual learners, students in immersion programs, or those from different sociocultural regions, would clarify whether the identified relationships hold under varied linguistic and instructional conditions.

Fifth, although temporal separation was built into the three-wave design, each interval spanned approximately 6 weeks within a single semester. This timeframe may capture short-term changes but might not reflect long-term motivational or affective development. Future longitudinal research should extend the observation window to multi-semester or academic-year durations, allowing examination of dynamic changes in self-efficacy, strategy use, and anxiety over time, as well as potential reciprocal influences among these constructs.

Finally, future work should also explore the moderating roles of contextual and individual factors, such as gender, writing experience, or instructional environment, which may influence how learners regulate emotions and strategies during writing. Integrating learning analytics, AI-assisted writing tools, or process-tracing methodologies (e.g., eye-tracking, screen-capture data) could yield finer-grained insights into the real-time cognitive and emotional mechanisms underlying writing performance. Such approaches would contribute to a more nuanced understanding of how self-efficacy, regulation, and anxiety interact dynamically within authentic learning environments.

## Data Availability

The raw data supporting the conclusions of this article will be made available by the authors, without undue reservation.
